# Attention to bone health in follow-up of gynaecological cancers in tertiary care

**DOI:** 10.1177/17455065211070747

**Published:** 2022-01-07

**Authors:** Catherine A O’Gorman, Sorcha Minnock, Joseph Mulhall, Noreen Gleeson

**Affiliations:** 1 Department of Gynecological Oncology, St. James’ Hospital, Dublin, Ireland; 2 Department of Gynecology, School of Medicine, Trinity College Dublin, Dublin, Ireland

**Keywords:** bone-health, cancer, gynaecological, menopause, osteoporosis, survivorship

## Abstract

**Objective:**

Women with gynaecological cancers are at an increased risk of cancer treatment–induced bone loss, which impacts on their quality of life and overall survival. Clinical cancer follow-up reviews focus on cancer status and fail to attend to important health and quality-of-life issues. We questioned whether there was a care-gap between tertiary clinicians and primary care physicians in the management of bone health in this cohort. Significant care-gaps in relation to bone health have been demonstrated in other oncologic settings. The objective of this study was to determine the level of attention to bone health in the care of women living with and beyond gynaecological cancer at a tertiary referral centre for gynaecological oncology.

**Methods:**

Retrospective, observational cohort study of attention to bone health in the management and follow-up of gynaecological cancers.

**Results:**

This study shows that there has been suboptimal attention from the carers at a cancer centre to bone health during the oncological follow up of women undergoing treatment for gynaecological cancer. In those at particular risk of cancer treatment–induced bone loss (iatrogenic menopause and/or external beam pelvic radiotherapy), 52% of women had no reference to bone health in their notes, and 57% had no assessment of bone mineral density.

**Conclusion:**

Tertiary cancer carers may underestimate the importance of bone health or believe that it falls outside the remit of their gynaecologic oncology service. Further research is needed to explore whether these findings are indicative of a true care gap and to gain insight into possible corrective measures.

## Introduction

Women with gynaecological cancers are at an increased risk of cancer treatment–induced bone loss (CTIBL) which may impact on their quality of life and overall survival.^1,2^ Diagnostic tests for loss of bone mineral density (BMD) are simple and non-invasive and there are effective therapies available to manage this condition. There is guidance available from national and international bodies on mitigation of CTIBL, but data from other oncological specialities where this is a survivorship issue demonstrate the presence of a care gap in this regard.^3^ We questioned whether there was a care gap between tertiary clinicians and primary care physicians in the management of bone health for women with gynaecological cancers.

The current European Society for Medical Oncology’s (ESMO) clinical practice guideline on bone health in cancer patients was published in 2020.^4^ Baseline evaluation of areal BMD (aBMD, g/cm^2^) with dual-energy X-ray absorptiometry (DXA) is advised for all women undergoing treatment known to accelerate loss of BMD. Risk factors include ovarian suppression, oophorectomy, or aromatase inhibitor (AI) therapy. Evaluation of risk of fracture through clinical risk factor assessment, with or without use of FRAX or other fracture probability prediction tool, is advised.^4^ Previously, in 2019, the American Society for Clinical Oncology (ASCO) published their clinical practice guideline on CTIBL.^5^ The expert panel advises that patients undergoing therapies with CTIBL potential or those with BMD or FRAX probability scores near treatment thresholds should have quantitative BMD assessment at 2 yearly maximum intervals. Both ESMO and ASCO strongly recommend that all persons at risk of CTIBL should have an assay of calcium and Vitamin D levels and be advised to consume a calcium-enriched diet with daily supplementary vitamin D. The B-ABLE study demonstrated that Vitamin D supplementation significantly reduces CTIBL.^4,6–8^

Harrington characterized osteoporosis care as ‘. . .the Bermuda Triangle made up of orthopaedists, primary care physicians and osteoporosis experts into which the fracture patient disappears’. . .^9^ We considered this could apply as well to the prevention of CTIBL. Primary care physicians for women with gynaecological cancers may expect this area of care is being catered for by secondary and tertiary services, while hospital carers may regard bone health as belonging to primary care. This potential gap in treatment was explored by Harvey et al.^3^ in *Mind the (treatment) gap: a global perspective on current and future strategies for prevention of fragility fractures*, which reviews guidance and the few studies looking at secondary- and tertiary-care physicians’ practices in relation to treatment-associated BMD loss. While care of gynaecological cancer patients was not explored specifically, other cancer services with treatments that increase the risk of osteoporosis are discussed.^10–12^

Traditionally, women with gynaecological cancers are reviewed regularly by their gynaecological oncology team for up to 5 years following their cancer treatment. CTIBL-associated therapies give rise to an accelerated loss early after treatment. Surgical menopause, due to bilateral oophorectomy which is standard of care in management of ovarian, endometrial, and some cervical cancers, results in a rate of bone loss that is 2–3 times the rate of that seen at natural menopause at 2 years.^13^ Chemotherapy-induced ovarian failure induces a loss of 7.7% in the first year.^5^ A recent, large meta-analysis and meta-regression of almost 4000 patients showed that 14% of women developed pelvic insufficiency fractures (PIF) following external beam radiation therapy for gynaecological cancers.^14^ The combination of chemotherapy and radiotherapy used to treat cervical cancers not amenable to surgical resection is associated with an 8% loss of lumbar BMD.^15^ Early preventive action or identification of loss of BMD is crucial to optimize patients’ quality of life and reduce fragility fracture rates. Follow-up reviews with tertiary-care clinicians (gynaecological oncologists, medical oncologists, and radiation oncologists) may not routinely include a review of bone health and so it is possible that a valuable opportunity to mitigate CTIBL is being missed.

Our aim was to determine whether full attention is being given to bone health during follow-up by gynaecological oncology tertiary carers. We hypothesized that there is suboptimal attention to bone health in the tertiary care of women with gynaecological cancers. To confirm this hypothesis, we conducted a retrospective observational cohort study in a tertiary gynaecological cancer care centre seeking to provide an objective measurement of attention to CTIBL in this setting.

## Methods

This is a retrospective, observational cohort study of attention to bone health in the management and follow-up of gynaecological cancers at a tertiary referral centre for gynaecological oncology. The cohort of women whose care was reviewed were those diagnosed with a gynaecological malignancy at the Trinity St James’s Cancer Institute in Dublin, Ireland, within a period of 12 months (May 2012 to April 2013 inclusive). These women were identified from the gynaecological oncology multi-disciplinary team (MDT) database which holds all the demographic and treatment information for each woman cared for in our department.

Following diagnosis of a gynaecological malignancy, routine follow-up in the gynaecological oncology clinics in our institution is for 5 years. This cohort was selected on the basis that they would have completed their routine 5-year gynaecological oncology follow-up. We included women diagnosed with any gynaecological malignancy, but as the focus of the study is on follow-up care, those who received all follow-up in other centres and those who died within a year of diagnosis were excluded. The malignancies were of uterine corpus, cervix, tubal/ovary/peritoneal, and vulva with two undefined.

Demographic information including age, menopausal status, use of hormone replacement therapy (HRT), baseline body mass index (BMI), smoking history, alcohol intake, co-morbidities, tumour site of origin, histology, grade, International Federation of Gynaecology and Obstetrics (FIGO) cancer stage, surgical procedures, chemotherapy and radiotherapy treatments, and iatrogenic menopause was extracted from the database. This information was confirmed, and augmented, by review of the electronic patient record consisting of clinical notes and clinic letters. Information including level of mobility, specific co-morbidities (conditions of the thyroid, liver and kidneys, malabsorption conditions or risk factors for malabsorption), bone health history, and long-term steroid use was recorded. The attention paid to bone health was assessed by recording when and how many times bone health was referenced during their follow-up, whether specific bone health biochemical parameters were measured (including Calcium and Vitamin D), and whether aBMD was quantitatively assessed by DXA.

This study was granted ethical approval by the Research Ethics Committee of St James’s Hospital/Tallaght University Hospital (REC2018-09CA4). In keeping with current data protection regulations, the need for patient consent was waived, and data were collected in a Microsoft excel file and subsequently anonymized.

Data analysis was carried out using R. Logistic regression was used to explore the impact of demographic and disease factors in relation to bone health assessment. Age was analysed as a continuous variable. Iatrogenic menopause was not suitable for inclusion as an independent variable in the regressions, and so bivariate analyses were performed to assess its impact. Chi-square tests were used for all outcome variables except Vitamin D measurement, where Fisher’s exact test was used because one cell had expected value less than 5. Significance was set at p values ⩽0.05.

## Results

### Cohort

A total of 293 women were diagnosed with a gynaecological malignancy in this time period at our institution. Sixty-two women did not meet the inclusion criteria, 31 were followed up elsewhere, and 31 survived less than 1 year. The remaining 231 women were included. Follow-up was until 60 months or death if sooner, with a mean follow-up of 54 months (median: 60 months, interquartile range (IQR): 0). Their median age was 57 years (range: 15–92 years) at diagnosis. Demographic and clinical risk factor information is summarized in Table 1.

**Table 1.** Demographics and clinical risk factors.

	Total cohort (n = 231)		Total cohort (n = 231)
Age		Menopausal status	
Median (range)	57 (15–92)	Premenopausal	73 (31.6%)
		Post-menopausal	155 (67.1%)
		Undetermined	3 (1.3%)
^a^Smoking status		Mobility status	
Current smokers	54 (25.4%)	Normal	208 (90%)
Ex-Smokers	43 (20%)	Reduced, no aid	18 (8%)
Never-smokers	116 (54.5%)	Reduced, uses aid	5 (2%)
^b^BMI		Secondary causes of reduced BMD	
Median (range)	28 (15–55)	None	198 (85.7%)
<18.5	5 (2.5%)	Hyperparathyroidism	2 (0.8%)
18.5–24.9	69 (34.2%)	Hyperthyroidism	2 (0.8%)
25–34.9	90 (44.6%)	Vit. D deficiency/ insufficiency	7 (3%)
⩾35	38 (18.8%)	Malabsorption	12 (5%)
		Liver disease	7 (3%)
		Glucocorticoids (>3/12)	3 (1.2%)

BMI: body mass index; BMD: bone mineral density.

a Smoking status recorded for 213 women only.

b BMI recorded for 202 women only.

Cancer sites, stage, and histopathological type are shown in Table 2. The malignancies were of uterine corpus (85/36.8%), cervix (55/23.8%), tubal/ovary/peritoneal (78/33.8%), vulva (11/4.8%), and others (2/0.8%).

**Table 2.** Cancer sites^a^, stages, and histopathological subtypes.

	Uterine (n = 85, 36.8%)	Tubal/ovary/peritoneal (n = 78, 33.8%)	Cervix (n = 55, 23.8%)	Vulva (n = 11, 4.8%)
Stage I	64 (75.3%)	Ia/b: 26 (33.3%) Ic: 13 (16.7%)	Ia/b1: 23 (41.8%) Ib2: 6 (10.9%)	4 (36.4%)
Stage II	2 (2.4%)	2 (2.6%)	10 (18.2%)	0 (0.0%)
Stage III	12 (14.1%)	28 (35.9%)	10 (18.2%)	5 (45.4%)
Stage IV	5 (5.9%)	9 (11.5%)	6 (10.9%)	2 (18.2%)
Subtype	Endometrioid 70 (82.4%) HGSC: 7 (8.2%) Clear cell: 2 (2.4%) Carcinosarcoma: 2 (2.4%) ^b^Others 4 (4.8%)	HGSC: 23 (33.3%) Borderline: 17 (21.5%) Endometrioid: 8 (10.4%) Mucinous: 6 (7.8%) Clear cell: 5 (6.4%) SCST: 5 (6.4%) ^c^Others 11 (14.1%)	SCC: 45 (81.8%) AdenoCa: 7 (12.8%) ASC: 2 (3.6%) Glassy Cell: 1 (1.8%)	SCC:11(100%)

HGSC: High Grade Serous Carcinoma; SCST: Sex Cord Stromal Tumour; ASC: Adenosquamous Carcinoma; SCC: Squamous Cell Carcinoma; AdenoCa: Adenocarcinoma.

a Not included are two retroperitoneal tumours: epithelioid malignant mesothelioma and retroperitoneal leiomyosarcoma.

b Mixed adeno-squamous tumour with neuroendocrine differentiation (n = 1), salivary duct type carcinoma (n = 1), leiomyosarcoma (n = 1), and undetermined (n = 1).

c Unspecified adenocarcinoma (n = 5), low-grade serous carcinoma (n = 2), carcinosarcoma (n = 1), germ cell tumour (n = 1), leiomyosarcoma (n = 1), and mixed mullerian (n = 1).

One hundred and sixty-two (70%) underwent bilateral oophorectomy as part of their cancer treatment, and of these, 39 (24%) were premenopausal and therefore had iatrogenic surgical menopause, 121 (75%) were postmenopausal, and 2 had undetermined menopausal status at age 49 years and 53 years. The median age of premenopausal women who underwent surgical menopause was 45 years (range: 17–55 years). Post-menopausal women who underwent bilateral oophorectomy had a median age of 63 years (range: 48–92 years), and of these, two were aged below 50 years, 48 were aged 50–60 years, 42 aged 60–70 years, and 29 were aged above 70 years. Radiotherapy was administered to 86 patients; 18 received vaginal vault brachytherapy (vvBT) alone and 68 women (29.4% of total cohort, median age = 56 years, range 21–88 years) received external beam radiotherapy either alone or in combination with vvBT. Chemotherapy was administered to 103 patients (median age = 57 years, range: 17–92 years); 32 of whom were premenopausal. A combination of chemo/radiotherapy was administered to 45 patients, of whom 22 were pre-menopausal. Rates of cancer treatment modalities related to bone loss within the total cohort are summarized in Table 3, and those resulting in an iatrogenic menopause are listed in Table 4.

**Table 3.** Rates of cancer treatment modalities related to bone loss within the total cohort (TC).

	Bilateral oophorectomy (BO), N = 162 (70% of total cohort)	External beam radiotherapy (EBRT), N = 68 (29% of total cohort)	Chemotherapy, N **=** 103 (45% of total cohort)
Bilateral oophorectomy	83 (36% of TC)	18 (7.8% of TC)	48 (20.8% of TC)
EBRT	18 (7.8% of TC)	5 (2.2% of TC)	32 (13.9% of TC)
Chemotherapy	48 (20.8% of TC)	32 (13.9% of TC)	10 (4.3% of TC)
BO, EBRT, and chemotherapy	13 (5.6% of TC)	13 (5.6% of TC)	13 (5.6% of TC)
*Total*	*162 total*	*68 total*	*103 total*

**Table 4.** Cancer treatments and iatrogenic menopause.

	N = 50 (%)
Bilateral oophorectomy (BO)	19 (38%)
External beam radiotherapy (EBRT) and BO	5 (10%)
Chemotherapy and BO	11 (22%)
Chemotherapy and EBRT	11 (22%)
BO, EBRT, and chemotherapy	4 (8%)

Fifty women underwent iatrogenic menopause (secondary to any treatment modality), and of these 10 were aged 50–55 years, 12 aged 45–49 years, 14 aged 40–44 years, 8 aged 30–39 years, 5 aged 20–29 years, and 1 aged under 20 years.

In relation to conditions strongly associated with secondary osteoporosis, two patients had hyperparathyroidism, and two had controlled hyperthyroidism. On first attendance to the gynaecological oncology clinic, five patients (2.7%) had a known Vitamin D deficiency, and two had an insufficiency. Three women (1.2%) had conditions linked to malabsorption (Coeliac disease, Crohn’s disease, and chronic high output ileostomy) and nine women (3.8%) had small bowel resections (one with residual small bowel length of 1 m). That left 220 (95%) without a known condition linked to malabsorption. Two of these 220 women had BMIs under 17 with actual weights of 37–42 kg. There were seven women (3%) with a chronic liver disease; three (1.2%) with haemochromatosis, 3 (1.2%) with liver metastases, and one with chronic hepatitis C. Three (1.2%) women had a significant history of glucocorticoid use (5 mg or more of prednisolone for at least 3 months).

### Attention to bone health

Findings in relation to attention to bone health are summarized in Table 5. There was no reference to bone health in the clinical letters or electronic patient record, during 5 years of clinical follow-up, for 148 (64%) of the cohort. Seventeen (7.4%) women had serum vitamin D measured and nine (53%) had a deficiency or insufficiency of vitamin D. Calcium levels were checked during follow-up for 40.7% of these women (n = 94). Vitamin D and calcium supplementation (or recommendation to start) was documented in 47 (20.4%) case notes.

**Table 5.** Attention to bone health.

	Total cohort (n = 231)	Iatrogenic menopause (IM) (n = 50)	External beam radiotherapy (EBRT) (n = 68)	IM and EBRT (n = 98)
Bone health referenced	83 (36%)	27 (54%)	32 (47%)	47 (48%)
DXA performed	57 (24.7%)	27 (54%)	27 (40%)	42 (43%)
Vitamin D levels checked	17 (7.4%)	3 (6%)	9 (13%)	12 (12.2%)
Vitamin D supplements	47 (20.4%)	12 (24%)	18 (28%)	23 (23.5%)
Calcium levels checked	94 (40.7%)	22 (44%)	44 (64%)	53 (54%)
Calcium supplements	47 (20.4%)	12 (24%)	19 (28%)	28 (28.6%)

DXA: dual-energy X-ray absorptiometry.

Quantitative assessment of aBMD was performed through DXA for 57 (24.7%) women. Osteoporosis/vertebral fractures and osteopenia were diagnosed in 13 (22.8%) and 18 (31.6%), respectively. Of all, 35 women (15%) had a DXA within 2 years following diagnosis, and 8 (23%) of these underwent repeat DXA scan within the following 3 years. Of these 8 women, 7 women meet criteria for treatment/prophylaxis with bone-modifying agents (BMAs). However, we see a deterioration of BMD to osteoporotic levels in 3 women, none of whom were taking BMAs (all of whom would meet the extrapolated ESMO criteria for prophylaxis), and another 3 women who remained osteoporotic, with only one of them taking BMAs.

Of the women who underwent iatrogenic menopause (n = 50), twenty-three (46%) had no reference to bone health in their subsequent clinical notes. These women (n = 23) ranged in age from 26 to 55 years (median age: 42 years). Of the women, 46% with iatrogenic menopause did not have a DXA scan to quantify aBMD during their 5-year follow-up (median age: 43 years, range: 25–55 years). Three (6%) women had their vitamin D level checked, and 22 (44%) had calcium levels checked during follow-up. Vitamin D and Calcium supplements were documented as taken by, or prescribed for, 12 (24%) women following iatrogenic menopause. Eight (16%) women were prescribed HRT following an iatrogenic menopause, with one declining therapy. Two (4%) women who underwent iatrogenic menopause were commenced on bisphosphonate therapy following a diagnosis of osteoporosis on DXA. Thirteen (26%) women with iatrogenic menopause met the ESMO criteria for prophylactic anti-resorptive therapy but were not taking BMAs.

Sixty-eight women (29.4%) underwent external beam radiotherapy. There was no reference to bone health documented in 36 (53%) case notes and 41 (60%) did not undergo densitometric bone density assessment with DXA. In all, 9 (13%) and 44 (64%) had their vitamin D and calcium levels checked, respectively, and supplementation documented as prescribed or used by 19 (28%).

When we combined these CTIBL-at-risk sub-groups (iatrogenic menopause and/or external beam radiotherapy, n = 98) we found that 51 (52%) of women had no reference to bone health in their notes, 56 (57%) had no BMD assessment, 85 (87.8%) had no measure of serum vitamin D, and 45 (46%) had no measure of calcium during follow-up. Seventy-five (76.5%) were not taking vitamin D supplementation.

In all, 43 women (19%) had documented low BMD in their medical history prior to cancer diagnosis or diagnosed during follow-up. Of these women, 34 (79%) had DXA and 7 (16%) were assessed for vitamin D deficiency. Thirty-three (77%) were taking calcium supplements, 29 (67%) were taking Vitamin D supplements, and 11 (26%) were taking antiresorptive medications. Reduced mobility lead to increased reference to bone health in the clinical notes (65% versus 36% of total cohort) and an increase in the rate of vitamin D assessment (17.4% versus 7.4% of total cohort); however, there was no substantial increase in the rate of DXA (26%).

Logistic regressions and bivariate analyses were conducted to assess the influence of patient and disease characteristics on four dependant variables, reference to bone health in clinical notes, measurement of calcium and vitamin D levels, and performance of DXA. Treatment with external-beam radiotherapy was positively associated with attention to bone health in three of the regressions, reference to bone health in clinical notes (odds ratio (OR) = 3.811, p = 0.002), Vitamin D measurement (OR = 4.607, p = 0.043), and performance of DXA (OR = 4.555, p = 0.002). Pre-menopausal status at diagnosis was positively associated with performance of DXA (OR = 4.190, p = 0.012), while older age (OR = 1.042, p = 0.016) and post-menopausal status (OR = 3.572, p = 0.017) were associated with calcium measurement. Chemotherapy was negatively associated with DXA performance (OR = 0.372, p = 0.027) and with bone health reference in clinical notes (OR = 0.391, p = 0.013). Logistic regression results are summarized in Table 6.

**Table 6.** Demographic and disease factors and in relation to bone health assessment.

	Bone health reference odds ratio (CI, p value)	DEXA scan odds ratio (CI, p value)	Vitamin D measured odds ratio (CI, p value)	Calcium measured odds ratio (CI, p value)
*Complete oophorectomy*	**OR = 1.104** CI: 0.521, 2.373 p = 0.798	**OR = 2.416** CI: 0.999, 6.248 p = 0.058	**OR = 1.620** CI: 0.412, 7.594 p = 0.513	**OR= 1.311** CI: 0.606, 2.815 p = 0.487
*External-beam radiotherapy*	OR = 3.811* CI: 1.675, 9.021 p = 0.002	OR = 4.555* CI: 1.763, 12.165 p = 0.002	OR = 4.607* CI: 1.053, 21.762 p = 0.043	**OR = 1.727** CI: 0.749, 4.145 p = 0.208
*Chemotherapy*	OR = 0.391* CI: 0.185, 0.810 p = 0.013	OR = 0.372* CI: 0.152, 0.883 p = 0.027	**OR = 1.626** CI: 0.420, 7.004 p = 0.494	**OR = 1.475** CI: 0.697, 3.128 p = 0.308
*Pre-menopausal at diagnosis*	**OR = 1.875** CI: 0.713, 4.959 p = 0.202	OR = 4.190* CI: 1.389, 13.079 p = 0.012	**OR = 1.020** CI: 0.134, 6.773 p = 0.984	OR = 3.572* CI: 1.282, 10.480 p = 0.017
*Current smoker*	**OR = 1.328** CI: 0.635, 2.763 p = 0.448	**OR = 0.887** CI: 0.374, 2.033 p = 0.780	**OR = 0.894** CI: 0.169, 3.789 p = 0.884	**OR = 0.959** CI: 0.455, 2.054 p = 0.913
*Increasing age*	**OR = 1.018** CI: 0.987, 1.050 p = 0.250	**OR = 1.006** CI: 0.969, 1.044 p = 0.752	**OR = 1.040** CI: 0.981, 1.102 p = 0.178	OR = 1.042* CI: 1.008, 1.078 p = 0.016

CI: confidence interval; OR: odds ratio.

* Denotes values meeting statistical significance.

Bivariate analyses demonstrate significant positive associations between iatrogenic menopause and attention to bone health. Clinical notes contained references to bone health in 54% of those with iatrogenic menopause versus 31% of those without (p = 0.004). Iatrogenic menopause was also positively related to the performance of DXA (54% versus 16.6%, p < 0.001). Calcium was more frequently assessed in those with iatrogenic menopause (80% versus 63%, p = 0.037) but Vitamin D measurement was not positively related to iatrogenic menopause (p = 1.000).

## Discussion

Many women are living years with and beyond gynaecological cancer and so our focus in follow-up must shift from survivorship alone to quality of health and all aspects of well woman care. More than 20% of women aged 50–84 years have osteoporosis and there is evidence to suggest that women with gynaecological malignancies, with the exception of endometrial cancer, have significantly lower BMD even prior to commencing cancer treatments compared to healthy controls.^16–18^ Only a quarter of our patients underwent DXA, and of those, more than one in two had bone density below the normal range. Along with a potential lower baseline BMD, research has demonstrated without doubt that treatments for gynaecological cancers are associated with loss of bone density. Surgical menopause results in an accelerated decline in BMD (compared to rate at natural menopause) from an earlier, potentially lower peak bone mass.^13^ A recent prospective cohort study of women undergoing pelvic radiotherapy for gynaecological cancers (with or without chemotherapy) demonstrated an increase in the rates of diagnosis of osteopenia/osteoporosis from 50% at baseline to 70% 2 years following treatment.^19^

A recent, large study of adults over 50 years of age in Ireland demonstrated vitamin D deficiency, defined as 25(OH)D concentration <30 nmol/L in 13% of participants.^20^ Clinical practice guidelines advise a target concentration of >75 nmol/L for protection of bone health.^20,21^ We found vitamin D was rarely measured in women in follow-up of gynaecological malignancies and serum levels were insufficient in more than half of those who had an assay. The sample size of those with measured vitamin D levels in our study is too small to draw conclusions in this regard but does highlight the lack of attention to this remediable condition. In addition to the key function of vitamin D in bone homeostasis, deficiency has been shown to be associated with increased rates of cancer and potentially reduced overall survival.^22–25^

There is no detailed guidance for bone health care in relation to gynaecological cancer, but ESMO and ASCO strongly recommend that all patients at risk of CTIBL in addition to quantitative BMD assessment should have calcium and vitamin D levels checked, consume a calcium-enriched diet, and daily supplementary vitamin D. These measures reduce CTIBL; however, only one in five of our patients were taking calcium and vitamin D supplements.^4,5,7,8,26^ Serum calcium measurement was performed more frequently than vitamin D levels. If the intention was to assess bone health parameters, one would expect vitamin D assessment rates to be more useful because serum calcium does not usually change with calcium reabsorption from bone in osteoporosis. This discrepancy is likely due to calcium assays forming part of routine oncology blood panels, and we do not regard serum calcium assays as indicative of attention to bone health in this cohort.

The majority of patients undergoing treatment of gynaecological cancers lose gonadal function if not already menopausal. Our cohort had a median age of 57 years. Two-thirds were postmenopausal and almost one-third were pre- or perimenopausal, so the majority would have been hypo-oestrogenic before or rendered so by their treatment. We limited our study to women surviving more than a year in order to exclude those with progressive disease for whom secondary sequelae of cancer treatment would be subordinate.

We hypothesized that there is suboptimal attention to bone health in the tertiary care of women with gynaecological cancers. To our knowledge, this is the first study to examine the provision of this aspect of survivorship care in the gynaecological oncology setting. Our results show a substantial lack of attention to bone health, even in the highest risk sub-groups. Within the subgroup of women who underwent an iatrogenic surgical menopause and/or those who received external beam radiotherapy, we found that more than half of the women had no reference to bone health in their clinical letters or electronic patient records and no BMD assessment performed during the 5 years of clinical follow-up. Vitamin D levels were not assessed for 88%, and over three quarters (77%) of this group were not taking any vitamin D supplement.

We acknowledge that practice demonstrated in this study is not in line with international recommendations which stipulate that all women who are to undergo a cancer treatment associated with accelerated loss of BMD should have their bone health status assessed at baseline. Recommended investigations include quantitative aBMD assessment with DXA, assessment of clinical risk factors and serum levels of calcium and Vitamin D at a minimum.^4,5^ Advice regarding modifiable risk factors (e.g. tobacco use, alcohol consumption) should be given along with lifestyle modifications such as increased exercise: weight bearing, balance, and posture exercises. Commencement of BMAs in certain scenarios is appropriate as a preventive measure, before traditional treatment thresholds are met.^4,5^ ESMO provides guidance on prophylactic BMAs based on hypo-oestrogenism and clinic-demographic factors. This is not focused on gynaecological cancers; however, the aetiological factors are similar – though without the added high-risk gynaecological factor of pelvic radiotherapy. In patients with hypo-oestrogenism due to oophorectomy or ovarian suppression therapy for breast or prostate cancer, ESMO recommend treatment or prophylaxis with BMAs if BMD T-score is less than −2.0, or two or more of the stated risk factors are present. These clinical risk factors include age greater than 65 years, T-score less than −1.5, ever smoker, BMI less than 24, family history of hip fracture, personal history of fragility fracture after age 50 years, and glucocorticoid use for 6months or more. One in four women who met the extrapolated ESMO criteria for prophylactic anti-resorptive therapy were not commenced on BMAs.^4^

Attention to bone health varied substantially between patient groups. Although attention to bone health was substantially lacking in all groups, the sub-groups at highest risk of CTIBL did receive relatively more attention. Women who received external beam radiotherapy were significantly more likely to have an assessment of bone health and pre-menopausal status at diagnosis and iatrogenic menopause were positively associated with DXA performance. Women who had a DXA performed were more likely to have had their vitamin D levels measured (n = 9, 16%) than the total cohort (7.4%). In keeping with our multi-variate analysis, two-thirds of these women received external beam radiotherapy. There was no significant association between older age and attention to bone health, and the cohort of women undergoing chemotherapy were significantly less likely to undergo bone health assessments. The median age of women undergoing chemotherapy was 57 years (IQR: 16.5 years) and two-thirds were post-menopausal and so they were neither older nor more likely to be already menopausal at diagnosis compared to the total cohort. The constituent cancer sites of this group were cervix (n = 35, 34%), uterine (n = 13, 13%), ovary (n = 54, 52%), and vulva (n = 1, 1%). The factors leading to reduced bone screening in this cohort are unclear. Perhaps this group, being 81% post-menopausal at diagnosis and 56% not undergoing radiotherapy appeared to the clinicians to be of lower risk for CTIBL. Post-menopausal women in this group merit attention to bone health given their already hypo-oestrogenic state and the potential direct toxicity that cytotoxic chemotherapy and its supportive therapies (steroids and proton pump inhibitors) exert on bone metabolism.^27,28^ Smoking, as a risk factor for osteoporosis, was not significantly associated with attention to bone health.

Clinical cancer follow-up reviews focus on cancer status and fail to attend to important health and quality of life issues.^29,30^ Our findings are not exclusive. While no prior studies have investigated the presence of a care-gap in the provision of bone health care in the gynaecological oncology setting, significant care-gaps in relation to bone health have been demonstrated in other oncological settings.^3^ Up to one-third of patients with prostate cancer receive androgen deprivation therapy (ADT) which may cause osteoporosis.^31^ There are guidelines in relation to CTIBL in prostate cancer,^4,32^ but a substantial care-gap exists.^11,12,33^ A Canadian survey of urologists and genitourinary radiation oncologists reported that while almost 70% were familiar with guidelines on CTIBL in prostate cancer patients receiving ADT, just one-third routinely assessed BMD as recommended. Less than 5% used a fracture risk assessment tool.^11^ A US study showed that just 8.6% of men with prostate cancer underwent BMD testing as recommended.^12^

Similarly, a care-gap for women receiving AI therapy for breast cancer was demonstrated. In reducing endogenous oestrogen production through aromatization of androgens, AI therapy may result in reduced BMD and fragility fractures. International guidance recommends baseline assessment of fracture risk with BMD assessment, biochemical assessment, and evaluation of clinical risk with a validated tool such as FRAX.^4,34^ A US study showed just 44% of women underwent BMD assessment as recommended on commencement of AI therapy, and 75% and 66% did not have follow-up BMD assessments at 2 years or 3 years post-initiation of therapy.^10^ There are no studies on this care-gap in the gynaecological oncology setting to serve as a comparison for our results. However, our findings are in line with the reported literature, briefly described above, from other survivorship settings.

The existence of these care-gaps is likely multifactorial and may include a lack of awareness or knowledge pertaining to CTIBL or perception that bone health falls within the primary care remit. System factors such as time constraints and other aspects of care competing for attention, funding, or access to investigations/therapies will impact on service provision. Perception of bone health as low priority has been demonstrated in qualitative studies in primary care among general practitioners, so tertiary clinicians should not assume that bone health care is being provided in the community.^35,36^ Communication between tertiary and primary care clinicians should include a clear plan as to whose responsibility it is to mitigate the detrimental effect cancer therapies may have on bone health. An inclusive survivorship plan, such as that mandated by the American College of Surgeons Commission on Cancer, is recommended.^5,37^

Inattention to bone health could have a detrimental effect on the general and cancer specific health of women. Studies have shown that frailty measures are predictive of risk of recurrence and overall survival in women with gynaecological cancers. Osteoporosis and osteopenia are associated with increased risk of all-cause mortality.^1^ Deterioration in bone health impacts negatively on quality of life. Fragility fractures, particularly those of the hip and vertebral bodies, result in pain with reduced mobility and function. PIFs occur in 14%–16% of women following external beam radiation therapy for gynecologic cancers.^14,38^ Osteoporosis, even in the absence of fractures, is associated with reduced health related quality of life.^2^

This is the first study to investigate the presence of a care-gap in CTIBL in gynaecological oncology. The highlighting of a previously unidentified deficit in care is valuable as it affords the opportunity to effect improvements in patient care. Traditionally women with gynaecological cancers were followed at their treating cancer centre for at least 5 years after treatment. This follow-up routine appears to have been focused on the detection of cancer recurrence and has failed to address many issues of women’s health including emotional, social, and sexual health.^29,30^ We want to emphasize that bone health also appears to be a casualty of the traditional care system. New paradigms are evolving that will shift intermediate and longer term care of women with gynaecological cancers away from this hospital based traditional follow-up.^39^ We strongly recommend that bone health is included in the new algorithms for surveillance of women after gynaecological cancer treatment.

There are limitations to this study. The sample size is modest and from a single centre. The study was designed as a retrospective observational study of the care provided to the cohort of patients treated in our cancer centre in a 12-month period, and over the course of their 60-month routine follow-up. Therefore, there was no formal power analysis, and as a retrospective study, it is subject to the possibility of incomplete data. We reviewed the patients’ electronic patient records, including clinical letters, laboratory results, and radiology, in full but we did not contact patients directly for information.

## Conclusion

This study shows that there has been suboptimal attention to bone health during the oncological follow-up at a tertiary-care cancer unit of women undergoing treatment for gynaecological cancer. This is similar to the care-gaps demonstrated in other oncological specialties. Tertiary cancer carers may underestimate the importance of bone health or believe that it falls outside the remit of their specialist oncology service. CTIBL can have a major impact on survivorship through its negative effect on quality of life and overall survival. Further research is needed to explore whether these findings are indicative of a true care-gap and how best to implement corrective measures.
